# The Impact of Community‐Based Midwife Continuity of Care Models for Women Living in Areas of Social Disadvantage and Ethnic Diversity in the United Kingdom: A Prospective Cohort Study

**DOI:** 10.1111/1471-0528.70101

**Published:** 2026-01-20

**Authors:** Cristina Fernandez Turienzo, Sam Burton, Zahra Khan, Hannah Rayment‐Jones, Mary Newburn, Paul T. Seed, Zoe Vowles, Jane Sandall, Abigail Easter, the eLIXIR‐BiSL Partnership, Lucilla Poston, Laura A. Magee, Robert Stewart, David Edwards, Mark Ashworth, Jane Sandall, Ingrid Wolfe, Cheryl Gillett, Michael Absoud, Lucy Pickard, Amanda Grey, Sarah Spring, Toyin Kazeem, Amelia Jewell, Matthew Broadbent, Finola Higgins, Leonardo de Jongh, Tisha Dasgupta, Carolyn Gill

**Affiliations:** ^1^ Department of Women and Children's Health, School of Life Course & Population Sciences, Faculty of Life Sciences & Medicine King's College London London UK; ^2^ School of Psychology Liverpool John Moores University Liverpool UK; ^3^ University of Doha for Science and Technology Doha Qatar

## Abstract

**Objective:**

Addressing inequalities in maternal and newborn health is a UK public health priority. Evidence on effective multi‐interventional strategies is urgently needed. This study evaluated the impact of community‐based midwife continuity of care (CBMCOC) models for women and babies in ethnically diverse and socially disadvantaged areas of South London.

**Design:**

We conducted a prospective cohort study using the eLIXIR, Born in South London, maternity–child data linkage.

**Setting:**

United Kingdom.

**Population:**

Pregnant women exposed to CBMCOC and standard care between 2018 and 2020.

**Methods:**

Propensity score matching (1:4) was used to account for differences between CBMCOC and standard care cohorts and control for confounding bias. Conditional logistic regression estimated risk ratios. Subgroup analysis included women of Black, Asian and other ethnic minority groups, and those living in highly deprived areas.

**Outcomes:**

The primary outcome was preterm birth (< 37 weeks' gestation). Secondary outcomes included other relevant maternal, perinatal, process and clinical variables.

**Results:**

Before matching, 12 386 women were exposed to standard care and 1338 to CBMCOC; after matching, 5352 and 1338 were included, respectively. The risk of preterm birth was lower among women exposed to CBMCOC (unmatched: 4.6% vs. 10.3%, RR = 0.50, 95% CI: 0.38–0.64; matched: 4.6% vs. 8.4%, RR = 0.54, 95% CI: 0.40–0.70). Subgroup analyses showed reduced preterm birth rates among ethnic minority women and those in deprived areas when exposed to CBMCOC.

**Conclusions:**

In this diverse population with a range of risk factors, locality‐based interventions integrating community‐based care and midwife continuity may reduce maternal and newborn health inequalities. Further trials of such models should be conducted.

## Abstract 2

**Linked article:** This article is commented on by Bone, pp. 974–975 in this issue. To view this article visit https://doi.org/10.1111/1471‐0528.70141.

## 1 Introduction

Global reports have described inequalities in maternal and newborn health outcomes and experiences for decades. Each year millions of women die from preventable causes that are not only related to complications in pregnancy, childbirth and the postnatal period, but also to manifestations of persistent global socioeconomic and health inequities [1]. The United Kingdom (UK) is no exception. Enquiries into maternal deaths have consistently found that women from the most disadvantaged and poorest backgrounds, and those from Black, Asian and minority ethnic groups are at greatest risk of severe mortality and morbidity [2, 3, 4, 5]. Their babies are also more likely to be stillborn, preterm, smaller than expected or die within their first month [3, 5, 6, 7, 8]. In addition, there are many challenges faced by recent migrants, and those with language barriers, emphasising the ongoing need for culturally sensitive care and improved access to services [7]. Integrative, holistic innovative solutions beyond maternity care are also needed to address underlying societal structures that impact health before, during and after pregnancy—such as housing, education and access to healthy environments [9, 10].

Three recent systematic reviews of policy, health and social care interventions to mitigate inequalities in maternal and newborn health among disadvantaged groups found that multicomponent approaches and interventions combining midwife continuity of care models and community‐based services could improve adverse pregnancy outcomes and increase access to care, attendance and engagement [11, 12, 13]. These findings are consistent with existing Cochrane reviews of community‐based intervention packages [14] and midwife continuity of care models [15], which highlight the need to integrate both maternal and neonatal care in community settings. These observations also align with health policy and the focus on people‐centred and place‐based models of maternity care that integrate health and care by shifting the way services are funded, managed and delivered ‘from health systems designed around diseases and health institutions towards health systems designed for people and communities’ [16, 17].

In England maternal policy includes an ambition to halve maternal mortality, neonatal mortality and serious brain injury in newborns by 2025 [18] and the Core20PLUS5 strategy to reduce inequalities in outcomes for mothers and babies [19]. There is some evidence on the effectiveness of continuity of care models (including place‐based care) for ‘at risk’ population groups, and limited understanding of care pathways and contextual factors surrounding severe mortality and morbidity among women with physical, mental and social risk factors [20, 21, 22]. We aimed to investigate the impact of community‐based midwife continuity of care models for women living in areas of ethnic diversity and social disadvantage in South London. To achieve this, we utilised a population maternal‐child data linkage of electronic health records, employing the method of propensity score matching to control for confounding bias.

## 2 Methods

### 2.1 Setting and Study Population

This prospective cohort study used the eLIXIR Born in South London (BiSL) programme [23]. eLIXIR is a unique population‐based database using opt‐out consent to collect real‐time, pseudonymised and routine maternity, neonatal and mental health data at two hospitals and primary care data from the Lambeth DataNet platform, enabling life course studies of physical and mental health in a large, diverse and inner urban population of South London, UK [23]. We included all pregnancies recorded between October 1, 2018 and March 1, 2020 (from the first antenatal appointment to discharge from maternity services). From an initial extraction of 13 795 pregnancies, we applied the following exclusion criteria: (1) multiple pregnancies (they wouldn't be eligible for community‐based midwife continuity of care but specialist care) and (2) pregnancies without complete data from the first antenatal appointment were excluded.

### 2.2 Data Sources

The eLIXIR Partnership database was utilised. Maternity and neonatal data for two maternity services are recorded on the BadgerNet electronic patient record system (CleverMed), which captures data from clinical records including clinical data, socio‐demographics, physical and mental health history, model of care and assessments for obstetric risk (e.g., a previous low birthweight or preterm infant, previous placental abruption or preeclampsia, gestational diabetes) and social risk (e.g., domestic violence, homelessness, drug misuse) [23]. Mental health data are obtained from the South London and Maudsley NHS Foundation Trust (SLaM) Clinical Records Interactive Search (CRIS) system, which generates variables extracted from electronic mental health records in SLaM. Primary care data are recorded from the Lambeth DataNet platform which captures, that is, clinical and development data, consultations, multimorbidity, long‐term conditions. For this study, relevant variables (detailed below) from BadgerNet systems, linked at the individual level as part of the eLIXIR partnership database, were extracted for the selected period, which represented the entire timespan available before the COVID‐19 pandemic and associated maternity services reconfiguration.

### 2.3 Exposure to Community‐Based Midwife Continuity of Care (CBMCOC) Models

There were eight models of CBMCOC in which the same midwife or team of midwives provided care to a woman from early pregnancy to the postnatal period; antenatal and postnatal care were predominantly provided in the community, and labour and birth care were at home or in the hospital. All models provided community‐based care based on women's geographical location and/or social vulnerability, and although many models were located in areas of high social deprivation, not all women under those models were considered vulnerable or had social risk factors. Some models included a team approach (a small team of midwives who share the caseload) while others included a caseload approach (a named and partner midwife who provide all the care), and the composition and modus operandi varied between the different models in terms of caseload size, team organization and on‐calls for childbirth care. However, in all models, midwives planned, organised and delivered comprehensive midwifery care in the community and hospital, that is, assessed needs, planned care, referred to other professionals, and coordinated services; they worked in partnership with the woman and with a multidisciplinary network of support. Women who developed complications in pregnancy, birth and the postnatal period were referred or escalated for obstetric care using the same clinical guidelines as in standard care; however the midwifery care continued to be provided from the team. Thus, while the models varied in composition and modus operandi, they were grouped together for the analysis. This approach was taken because all eight models shared the core principles of delivering midwife continuity of care within a community‐based setting, allowing for a sufficiently powered evaluation of this overall care type against standard care.

### 2.4 Exposure to Other Models of Maternity Care

Other models of maternity care included standard care models where obstetricians were the lead professionals for antenatal care and rostered midwives provided in‐hospital labour, birth and postpartum care for women having obstetrician‐led care (not necessarily by the obstetrician providing or leading antenatal care); and where midwives, GPs and obstetricians shared the responsibility for the organisation and delivery of care throughout the initial booking to the postnatal period provided in both hospital and/or community settings. These models are similar in that they do not aim to provide midwife continuity of care.

### 2.5 Outcomes

The primary outcome was preterm birth, defined as any birth that occurs before 37 completed weeks of gestation. Secondary maternal outcomes included onset of labour (spontaneous onset, induction, caesarean before labour), intrapartum analgesia/anaesthesia (none, epidural, spinal), mode of birth (spontaneous cephalic, vaginal breech, caesarean), section (elective and emergency) and instrumental (forceps and ventouse) birth, place of birth (hospital, home, other), perineal status after birth (intact perineum, first‐ and second‐degree tear, episiotomy, third‐ and fourth‐degree tear), estimated blood loss more than 500 mL, and maternal admission longer than 7 days. Secondary perinatal outcomes included stillbirth (born with no signs of life at or after 24 weeks of pregnancy) or neonatal death (death during the first 28 days), Apgar score at 5 min less than or equal to 7, low birthweight (< 2500 g), small for gestational age, skin‐to‐skin contact, first feed method (breast, bottle, other), admission to the neonatal unit. Secondary process outcomes included late booking for antenatal care (after 20 weeks), missed appointments, antenatal admissions (other than birth), and referrals (smoking, mental health and child protection). See Table S1 for BiSL variable definitions.

### 2.6 Co‐Variables

The following variables were adjusted for in all models: participants' socio‐demographic characteristics included maternal age, self‐reported ethnicity (grouped into: White, Black, Asian, Mixed and Other), Index of Multiple Deprivation quintile (IMD, a method used to measure social and economic deprivation in small areas of England and Wales; a score of 1 indicates the most deprived and a score of 5 the least deprived) [24]. Clinical and other characteristics at the first antenatal visit included: parity, previous preterm birth, existing physical or mental health conditions (including Whooley questions for identification of possible depression [25]).

### 2.7 Ethics Approvals

Ethical approval eLIXIR is granted by the Oxford Central Research Ethics Committee (23/SC/0116). The Health Research Authority Confidentiality Advisory Group (HRA CAG Ref: 18/CAG/0040) provided approval under Section 251 (s251) of the NHS Act (2006). This study was also approved by the eLIXIR Oversight Committee (RAF: DL021R).

### 2.8 Statistical Analysis

The sample size required was calculated based on the preterm birth rate with an expected 2% reduction aligned with the national maternity policy safety ambition policy [26]. We anticipated 240 women per year from each community‐based midwife continuity of care model and 2 years of data collection, meaning 1920 women exposed to community‐based models, and (with 4:1 matching) 7680 women exposed to standard care models, to provide 86.4% power to detect a reduction in preterm birth from 8% to 6%. Propensity scores were calculated for each woman using multivariable logistic regressions based upon the covariates: age, ethnicity, indices of deprivation (IMD), parity, social risk factors, Whooley positive, pre‐existing medical and mental conditions, along with an interaction term between Whooley positive and pre‐existing mental health condition. To ensure that all covariates were measured, a total of 4122 cases in which one covariate was unmeasured were removed from the sample, Data were deemed to be missing completely at random and therefore complete case analysis was adopted over multiple imputation [27]. Matching was performed in R using the packages, MatchIt [28], Optmatch [29] and MatchThem [30]. Nearest neighbour matching was used at a ratio of 1:4 and caliper of 0.2; given the much larger untreated group this ratio was chosen to reduce selection bias [31, 32]. Analysis included all women based on their assigned care model at the beginning of pregnancy, regardless of whether they fully adhered to that assigned model.

Demographic variables were compared using standardised mean differences (SMD) to assess differences in characteristics before and after the matching process (a SMD greater than 0.1, or 10% would indicate an imbalance). We fitted logistic regression models to compare key maternal outcomes (e.g., onset of labour, mode of birth) and perinatal outcomes (e.g., preterm birth, stillbirth, birthweight, Apgar score) between the two care groups, when the outcome was binary. Multinomial logistic regressions were fitted to investigate the impact of the intervention on non‐binary (multiple level) outcomes (e.g., perineal trauma, type of delivery, onset of birth), producing bootstrapped 95% confidence intervals. A sub‐group analysis by social deprivation and ethnicity was conducted, to assess the effect of the care model on preterm birth. Women from Black, Asian, mixed or other ethnic backgrounds were compared to women of White ethnicity. Women living in IMD quintile 1 or 2 were compared to those living in IMD quintiles 3, 4 and 5. Before and after propensity scoring models were also adjusted for age, ethnicity, deprivation, parity, prior preterm birth, social risk factors, Whooley positive result, pre‐existing medical and mental health conditions. To account for unmeasured confounding *E* values were calculated for all models. All models contained a random intercept of women's ID to account for shared variance across pregnancies. As recommended for sensitivity analysis for observational research [33, 34], we also calculated *E*‐values, which represent the strength of association that an unmeasured confounder would require with the exposure and outcome to attenuate main associations to non‐significance. R was used for data manipulation and analysis using the following packages, lme4 [35], tidyverse [36], naniar [37], jtools [38] and afex [39].

## 3 Results

17 917 pregnancies were initially extracted with birth outcomes; 4122 were excluded due to multiple pregnancies or missing data for the covariates of ethnicity and IMD from antenatal booking. Data relating to 13 795 pregnancies from 1 October 2018 to 1 March 2020 were finally extracted. 251 completed duplicates were removed from the dataset. The first pregnancy recorded within the eLIXIR database was considered the index pregnancy. Overall, 13 609 pregnancies with completed data from their first antenatal appointment were included in the final dataset and analysis.

### 3.1 Baseline Characteristics

The baseline characteristics of women exposed to standard care and women exposed to CBMCOC before propensity score matching (12 386 and 1338, respectively) and after matching (5352 and 1338, respectively) are presented in Table 1. Before matching, there were differences between women in CBMCOC and standard care in some sociodemographic and clinical characteristics. Women in CBMCOC were more likely to be primiparous, White and less likely to be Asian, more likely to be born in the UK and have English as their primary language compared to women in standard care. They were also more likely to have a Whooley positive and prior mental health condition, showing moderate imbalance in comparison to standard care. Following matching score adjustment, there were negligible imbalances between the groups.

**TABLE 1:** Maternal baseline characteristics.

Socio‐demographics						
	Unadjusted			Propensity‐adjusted		
	Standard care *n* = 12386	CBMCOC *n* = 1338	*SMD*	Standard care *n* = 5352	CBMCOC *n* = 1338	*SMD*
Age at booking (years)	32.71 (5.19)	32.73 (5.36)	0.00	32.81 (5.42)	32.73 (5.36)	0.01
Ethnicity^a^						
White	6352 (51.3)	778 (58.1)	0.14	3091 (57.7)	778 (58.1)	0.00
Black	2662 (21.5)	265 (19.8)	0.03	1116 (20.8)	265 (19.8)	0.03
Asian	1127 (9.1)	65 (4.9)	0.18	300 (5.6)	65 (4.9)	0.08
Mixed	561 (4.5)	78 (5.8)	0.09	280 (5.2)	78 (5.8)	0.06
Other	867 (7.0)	73 (5.4)	0.08	269 (5.0)	73 (5.5)	0.05
Not stated	817 (6.6)	79 (5.9)	0.03	296 (5.5)	79 (5.9)	0.02
IMD quintile						
1 (most deprived)	2424 (19.6)	263 (19.7)	0.01	1045 (19.5)	263 (19.7)	0.00
2	5123 (41.4)	567 (42.4)	0.01	2223 (41.5)	567 (42.4)	0.01
3	3156 (25.5)	369 (27.6)	0.03	1491 (27.8)	369 (27.6)	0.01
4	1225 (9.9)	99 (7.4)	0.09	414 (7.7)	99 (7.40)	0.02
5 (least deprived)	458 (3.6)	40 (3.0)	0.07	179 (3.3)	40 (3.0)	0.06
Born in the UK (yes)	5154 (41.6)	740 (55.3)	0.28	2883 (53.9)	740 (55.3)	0.02
Primary language English	8545 (69.0)	999 (74.7)	0.12	4064 (75.9)	999 (74.7)	0.01
Support status at booking^b^						
Supported	11114 (89.0)	1181 (88.3)	0.01	4848 (90.6)	1181 (88.3)	0.08
Unsupported	169 (1.4)	19 (1.4)	0.01	90 (1.7)	19 (1.4)	0.02
Sheltered accommodation	5 (0.0)	5 (0.4)	0.12	3 (0.1)	5 (0.4)	0.07
Other	144 (1.2)	23 (1.7)	0.15	85 (1.6)	23 (1.7)	0.05
Clinical and social characteristics						
Gestational age at booking (weeks)	12.08 (7.01)	11.00 (5.41)	0.17	11.06 (5.72)	11.00 (5.41)	0.01
BMI at booking	24.20 (6.37)	24.03 (6.15)	0.03	24.04 (6.16)	24.03 (6.15)	0.00
Primiparous	5732 (43.6)	793 (59.3)	0.26	3111 (58.13)	793 (59.3)	0.07
Smoking at booking	462 (3.7)	67 (5.0)	0.11	266 (5.0)	67 (5.0)	0.00
Pre‐existing physical conditions^c^						
Hypertension	193 (1.6)	7 (0.5)	0.08	80 (1.5)	7 (0.5)	0.08
Asthma	32 (0.3)	5 (0.4)	0.02	15 (0.3)	5 (0.4)	0.02
Autoimmune disease	151 (1.2)	10 (0.7)	0.04	55 (1.0)	10 (0.7)	0.03
Diabetes	273 (2.2)	10 (0.7)	0.12	101 (1.9)	10 (0.7)	0.09
Chronic renal disease	95 (0.8)	4 (0.3)	0.05	38 (0.7)	4 (0.3)	0.05
Haematology disorders	604 (4.88)	40 (2.9)	0.10	191 (3.6)	40 (2.9)	0.03
Cardiac disorders	52 (0.42)	6 (0.45)	0.00	16 (0.30)	6 (0.4)	0.00
Pre‐existing mental health conditions	2170 (17.5)	401 (30.0)	0.32	1676 (31.3)	401 (30.0)	0.02
Whooley Positive	977 (7.9)	243 (18.2)	0.37	934 (17.5)	243 (18.2)	0.02
Obstetric risk	1785 (14.4)	181 (13.5)	0.03	743 (13.9)	181 (13.5)	0.01
Previous preterm birth	346 (2.8)	49 (3.66)	0.05	192 (3.6)	49 (3.66)	0.03
Social risk	1424 (11.5)	172 (12.7)	0.04	3131 (58.5)	799 (59.7)	0.02

*Note:* Data are *n* (%), Age at booking and gestational age at booking: mean + standard deviation, BMI at booking: median (inter‐quartile range).

Abbreviations: BMI, body mass index; CBMCOC, community‐based midwife continuity of care; CI, confident interval; IMD, index of multiple deprivation; RR, risk ratio; SMD, standardised mean differences.

^a^ 817 in standard care models and 79 in community‐based models were missing, not recorded or not stated (propensity‐adjusted: 296 and 79 respectively).

^b^ 1123 in standard care models and 110 in community‐based models were missing (propensity‐adjusted: (propensity‐adjusted: 326 and 110 respectively)).

^c^ Poorly recorded and reported the most common pre‐existing conditions.

### 3.2 Primary Outcome

Women in CBMCOC experienced a significantly reduced risk of preterm birth compared to those in standard care. Before adjustment, preterm birth occurred in 4.6% of the CBMCOC group and 10.3% of the standard care group, corresponding to a risk ratio (RR) of 0.50 (95% CI: 0.38–0.64) and an absolute risk difference (RD) of −5.8 percentage points. After propensity score adjustment, the risk of preterm birth in CBMCOC remained significantly lower at 4.6% versus 8.4% in standard care (RR 0.54, 95% CI: 0.40–0.70; RD –3.8 pp). These results indicate that CBMCOC was associated with an absolute reduction of 38–58 fewer preterm births per 1000 women compared with standard care (Table 2).

**TABLE 2:** Primary outcome.

	Unadjusted						Propensity‐adjusted^a^					
	Standard care *n* = 12386	CBMCOC *n* = 1338	RR (95% CI)	RD	*p*	*E* value	Standard care *n* = 5352	CBMCOC *n* = 1338	RR (95% CI)	RD	*p*	*E* value
Preterm birth (< 37 weeks)	1277 (10.3)	61 (4.6)	0.50 (0.38, 0.64)	−5.7	< 0.001	3.41	450 (8.4)	61 (4.6)	0.54 (0.40, 0.70)	−3.8	< 0.001	3.11

*Note:* Data are *n* (%).

Abbreviations: CBMCOC, community‐based midwife continuity of care; CI, confident interval; RD, risk difference; RR, risk ratio.

^a^ Adjusted for age, ethnicity, deprivation Index quintile, parity, previous preterm birth, social risk factors, Whooley positive, pre‐existing medical and mental conditions.

### 3.3 Secondary Outcomes

Secondary maternal outcomes are presented in Table 3. After adjustments and compared to women in standard care, women in CBMCOC were more likely to experience spontaneous onset of labour (59% vs. 47% RD +12 pp), spontaneous vaginal birth (63% vs. 49%, RD +13 pp) and skin‐to‐skin contact after birth (85% vs. 79%, RR 1.09, 95% CI: 1.03–1.16; RD –6.0 pp); and they were less likely to use intrapartum analgesia/anaesthesia (14% vs. 9%; RR 1.68, 95% CI: 1.42–1.99; RD +5.6 pp) or experience induction of labour (24% vs. 27%; RR 0.71, 95% CI: 0.61–0.82, RD –2.6 pp), caesarean birth (25% vs. 36%; RR 0.53 95% CI: 0.46–0.61; RD 13.6 pp) and instrumental birth (11% vs. 14%; RR 0.65 95% CI: 0.53–0.79, RD –2.9 pp). In terms of perineal outcomes, CBMCOC women were less likely to have an episiotomy (12% vs. 16%, RR 0.71, 95% CI: 0.59, 0.85; RD –4.2 pp) and more likely to have first‐ or second‐degree tears (41% vs. 32%, RR 1.25, 95% CI: 1.08–1.44; RD +9.0 pp), with no differences in intact perineum and third‐ or fourth‐degree tears. The risk of blood loss over 500 mL was also reduced in CBMCOC (38% vs. 45%, RR 0.85, 95% CI: 0.77–0.93; RD –7.0 pp). Women exposed to CBMCOC were also more likely to give birth at home (15.7% vs. 0.9%; RR 17.41, 95% CI: 12.61–24.03; RD +14.8 pp), with corresponding reductions in hospital birth and prolonged postnatal stay in comparison to those in standard care (9% vs. 11%, RR 0.71, 95% CI: 0.57–0.87).

**TABLE 3:** Secondary maternal outcomes.

	Unadjusted						Propensity‐adjusted^a^					
	Standard care *n* = 12386	CBMCOC *n* = 1338	RR (95% CI)	*p*	RD	*E* value	Standard care *n* = 5352	CBMCOC *n* = 1338	RR (95% CI)	*p*	RD	*E* value
Onset of labour												
Spontaneous^b^	5669 (45.7)	787 (58.8)			13.1		2492 (46.6)	787 (58.8)			12.2	
Induction	3197 (25.8)	324 (24.2)	0.72 (0.63, 0.83)	< 0.001	−1.60	2.12	1432 (26.8)	324 (24.2)	0.71 (0.61, 0.82)	< 0.001	−2.6	2.17
Planned caesarean birth before onset of labour	3404 (27.5)	227 (17.0)	0.47 (0.40, 0.56)	< 0.001	−10.5	3.68	1428 (16.7)	227 (17.0)	0.49 (0.42, 0.58)	< 0.001	0.3	3.50
Analgesia/anaesthesia												
None^b^	1023 (8.3)	192 (14.4)	1.68 (1.43, 1.96)	< 0.001	6.1	2.75	466 (8.7)	192 (14.4)	1.68 (1.42, 1.99)	< 0.001	5.7	2.75
Epidural	3649 (29.5)	273 (20.4)			−9.1		1751 (32.7)	273 (20.40)			−12.31	
Spinal	2942 (23.7)	220 (16.4)			−7.3		1438 (26.9)	220 (16.4)			−10.5	
Type of birth												
Spontaneous Cephalic vaginal birth^b^	5705 (46.1)	844 (63.1)			17.0		2651 (49.5)	844 (63.1)			13.6	
Breech birth	65 (0.5)	7 (0.5)	0.82 (0.37, 1.80)	0.612	0.0	1.74	23 (0.4)	7 (0.5)	0.97 (0.41, 2.27)	0.937	0.1	1.21
Caesarean birth	4565 (36.8)	334 (25.0)	0.50 (0.44, 0.58)	< 0.001	17.2	3.41	1909 (35.7)	334 (25.0)	0.53 (0.46, 0.61)	< 0.001	13.6	3.18
Planned	2032 (16.4)	135 (10.1)			−6.3		883 (16.5)	135 (10.1)			−6.4	
Unplanned	2533 (20.4)	199 (14.9)			−5.5		1026 (19.2)	199 (14.9)			−4.3	
Instrumental birth	1930 (16.5)	153 (11.4)	0.61 (0.51, 0.74)	< 0.001	−5.1	2.66	766 (14.3)	153 (11.40)	0.65 (0.53, 0.79)	< 0.001	−2.9	2.45
Perineal status^c^												
Intact perineum^b^	3813 (30.8)	455 (34.0)			3.2		1766 (33.0)	455 (34.0)			1.0	
1^st^/2^nd^ degree tear	3943 (31.8)	548 (41.0)	1.21 (1.06, 1.38)	< 0.01	9.2	1.71	1711 (32.0)	548 (41.0)	1.25 (1.08, 1.44)	< 0.01	9.0	1.81
3^rd^/4^th^ degree tear	237 (1.9)	27 (2.0)	1.21 (0.80, 1.84)	0.364	0.1	1.71	83 (1.6)	27 (2.0)	1.34 (0.85, 2.10)	0.205	0.4	2.01
Not known	32 (0.3)	2 (0.1)					0 (0.0)	2 (0.1)				
Episiotomy	2265 (18.3)	163 (12.2)	0.62 (0.52, 0.73)	< 0.001	−6.1	2.61	877 (16.4)	163 (12.2)	0.71 (0.59, 0.85)	< 0.001	−4.2	2.17
Blood loss > 500 mls	5825 (47.0)	504 (37.7)	0.83 (0.75, 0.91)	< 0.001	−9.3	1.70	2391 (44.7)	504 (37.7)	0.85 (0.77, 0.93)	< 0.001	−7.0	1.63
Place of birth (where known)												34.31
Home	78 (0.6)	211 (15.7)	21.79 (16.80, 28.55)	< 0.001	15.1	43.07	46 (0.9)	211 (15.7)	17.41 (12.61, 24.03)	< 0.001	14.8	
Hospital	9162 (74.0)	853 (63.8)			−10.2		3987 (74.5)	853 (63.8)			−10.7	
Missing	3146 (25.4)	274 (20.5)					1327 (24.8)	274 (20.6)				
Skin to skin established after birth	10375 (83.8)	1251(79.3)	1.09 (1.03, 1.16)	0.003	−4.5	1.40	4566 (85.3)	1251(79.3)	1.09 (1.03, 1.16)	0.005	−6.0	1.40
Postnatal admission longer than 7 days	1409 (11.4)	118 (8.8)	0.73 (0.59, 0.88)	< 0.01	−2.6	2.17	601 (11.2)	118 (8.8)	0.71(0.57, 0.87)	< 0.001	−2.3	

*Note:* Data are *n* (%).

Abbreviations: CBMCOC, community‐based midwife continuity of care; CI, confident interval; RD, risk difference; RR, risk ratio.

^a^ Adjusted for age, ethnicity, deprivation Index quintile, parity, previous preterm birth, social risk factors, Whooley positive, pre‐existing medical and mental conditions.

^b^ Variable selected for conditional logistic regression.

^c^ 4361 in standard care models and 306 in community‐based models were missing or not recorded (propensity‐adjusted: 1845 and 306 respectively).

Perinatal outcomes are shown in Table 4. CBMCOC and standard care groups did not differ for the risk of stillbirth or neonatal death. The proportion of small for gestational age babies and low birthweight babies was significantly lower in CBMCOC compared to standard care (5.7% vs. 7.6%, RR 0.75, 95% CI: 0.59–0.95; RR −1.9 pp and 5.7% vs. 9.4%; RR 0.63, 95% CI: 0.50–0.39; RD –3.7 pp, respectively). There were neither differences in first feed method nor in admission to the neonatal unit, or five‐minute Apgar score.

**TABLE 4:** Secondary perinatal outcomes.

	Unadjusted						Propensity‐adjusted^a^					
	Standard care *n* = 12386	CBMCOC *n* = 1338	RR (95% CI)	*p*	RD	*E* value	Standard care *n* = 5352	CBMCOC *n* = 1338	RR (95% CI)	*p*	RD	*E* value
Birth outcome												
Livebirth^b^	12142 (98.0)	1336 (99.8)	5.82 (1.87, 30.76)	0.802	1.8	11.11	5313 (99.3)	1336 (99.8)	5.87 (1.85, 31.69)	0.852	0.5	3.11
Stillbirth or neonatal death	128 (1.0)	2 (0.1)			−0.4		39 (0.7)	2 (0.1)			−0.2	
Five minutes Apgar score > 7	11519 (93.0)	1279 (97.8)	1.27 (0.89, 1.87)	0.833	4.8	1.85	5040 (94.2)	1279 (97.8)	1.31 (0.90, 1.93)	0.831	3.6	1.45
Low birthweight (< 2.500g kg)	1240 (10.0)	76 (5.7)	0.57 (0.44, 0.72)	< 0.001	−4.3	2.90	501 (9.4)	76 (5.7)	0.60 (0.46, 0.75)	< 0.001	−3.7	2.72
Small for gestational age	1117 (9.0)	76 (5.7 )	0.63 (0.50, 0.79)	< 0.001	−3.3	2.78	406 (7.6)	76 (5.7)	0.75 (0.59, 0.95)	< 0.05	−1.9	2.50
First feed method			1.02 (0.97, 1.09)	0.362		1.16			1.03 (0.96, 1.09)	0.415		1.21
Breast^b^	10606 (85.6)	1222 (91.3)			5.7		4642 (86.7)	1222 (91.3)			4.6	
Bottle	986 (7.9)	83 (6.2)			−1.7		450 (8.4)	83 (6.2)			−2.2	
Other: NGT, etc	794 (6.4%)	33 (2.5)			−3.9		8 (0.1)	33 (2.5)			2.4	
Admission to the neonatal unit	964 (7.7)	73 (5.5)	1.40 (0.53, 3.72)	0.647	−2.2	6.87	427 (8.0)	73 (5.5)	1.45 (0.53, 3.97)	0.633	−2.5	5.33

*Note:* Data are *n* (%).

Abbreviations: CBMCOC, community‐based midwife continuity of care; CI, confident interval; NGT, nasogastric tube; RD, risk difference; RR, risk ratio.

^a^ Adjusted for age, ethnicity, deprivation Index quintile, parity, previous preterm birth, social risk factors, Whooley positive, pre‐existing medical and mental conditions.

^b^ Variable selected for conditional logistic regression.

Process outcomes (Table 5) showed no significant differences after adjustments in late booking for antenatal care (16% vs. 18%; RD –2.0 pp). Missed appointments were significantly lower in CBMCOC (5.4% vs. 15.7%; RR 0.66, 95% CI: 0.55–0.78; RD –10.3 pp) and antenatal admissions (excluding birth) were rare (< 1%) in both groups. Referral patterns differed; women in CBMCOC were more likely to receive referrals for mental health services (5% vs. 3%; RR 1.80, 95% CI: 1.26–2.37) with no differences in referrals for smoking or child protection.

**TABLE 5:** Secondary outcomes: processes.

	Unadjusted						Propensity‐adjusted^a^					
	Standard care *n* = 12386	CBMCOC *n* = 1338	RR 95% CI	*p*	RD	*E* value	Standard care *n* = 5352	CBMCOC *n* = 1338	RR 95% CI	*p*	RD	*E* value
Late booking for antenatal care	1870 (15.1)	211 (15.8)	0.99 (0.93, 1.05)	0.720	−0.7	1.11	954 (17.8)	211 (15.8)	0.99 (0.93, 1.06)	0.806	−2.0	1.11
Reason: (descriptive)												
Not letter/reminder	26 (1.4)	3 (1.4)					9 (0.9)	3 (1.4)				
Late referral	360 (19.3)	46 (21.8)					133 (13.9)	46 (21.8)				
Moved within the UK	162 (8.7)	22 (10.4)					48 (5.0)	22 (10.4)				
Appointment re‐scheduled	72 (3.6)	9 (4.3)					25 (2.6)	9 (4.3)				
Unaware/unsure of pregnancy/PV bleed	217 (11.6)	15 (7.1)					88 (9.2)	15 (7.1)				
Unwell/too sick to attend	5 (0.3)	0 (0.0)					2 (0.2)	0 (0.0)				
Other or not stated	1028 (55.1)	116 (55)					649 (68.0)	116 (55.0)				
One or more missing appointments	1085 (8.8)	71 (5.4)	0.66 (0.56, 0.79)	< 0.0001	−3.4	2.40	841 (15.7)	71 (5.4)	0.66 (0.55, 0.78)	< 0.001	−10.3	2.40
One or more antenatal admissions (other than birth)	63 (0.5)	2 (0.1)	0.27 (0.04, 0.88)	0.072	−0.4	6.87	23 (0.43)	2 (0.1)	0.34 (0.05, 1.15)	0.142	−0.3	5.33
Referrals												
Smoking	373 (3.0)	63 (4.7)	1.38 (1.04, 1.79)	< 0.05	1.7	2.10	210 (3.9)	63 (4.7)	1.14 (0.86, 1.51)	0.352	0.8	1.54
Mental health	234 (1.9)	70 (5.2)	2.44 (1.85, 3.19)	< 0.001	3.3	4.31	185 (3.5)	70 (5.2)	1.80 (1.26, 2.37)	< 0.001	1.7	3.00
Child protection	15 (0.1)	4 (0.3)	3.24 (0.89, 9.54)	< 0.05	0.2	5.93	11 (0.21)	4 (0.3)	2.51 (0.79, 7.97)	0.118	0.0	4.46

*Note:* Data are *n* (%). *n/N* (%) indicates that the denominator only includes participants with a relevant measurement for that variable.

Abbreviations: CBMCOC, community‐based midwife continuity of care; CI, confident interval; RD, risk difference; RR: risk ratio.

^a^ Adjusted for age, ethnicity, deprivation Index quintile, parity, previous preterm birth, social risk factors, Whooley positive, pre‐existing medical and mental conditions.

### 3.4 Subgroup Analysis

Subgroup analyses (Table 6) suggested that reductions in preterm birth in women in CBMCOC were evident across disadvantaged populations. Among women of Black, Asian, mixed, or other ethnicities, preterm birth was significantly reduced from 9.5% in standard care to 6.4% in CBMCOC (RR 0.66, 95% CI: 0.44–0.95; RD –3.1 pp) compared to women of Black, Asian, mixed, or other ethnicities exposed to standard care. Similarly, among women in the most deprived quintiles, preterm birth fell from 8.2% to 5.1% (RR 0.60, 95% CI: 0.43–0.82; RD –3.1 pp) compared to those in standard care.

**TABLE 6:** (A) Subgroup 1: Effect among Black, Asian, mixed and other ethnic groups of women on preterm birth and outcome. (B) Subgroup 2: Effect among women living in high deprivation (IMD 1 and 2) on preterm birth outcome.

(A)												
	Unadjusted						Propensity‐adjusted^a^					
	Standard care *n* = 5217	CBMCOC *n* = 481	RR 95% CI	*p*	RD	*E* value	Standard care *n* = 1965	CBMCOC *n* = 481	RR 95% CI	*p*	RD	*E* value
Preterm birth (< 37 weeks)	550 (10.5)	31 (6.4)	0.60 (0.41, 0.85)	< 0.01	−4.1	2.72	186 (9.5)	31 (6.4)	0.66 (0.44, 0.95)	< 0.05	−3.1	2.40

(B)												
	Unadjusted						Propensity‐adjusted^a^					
	Standard care *n* = 7447	CBMCOC *n* = 830	RR 95% CI	*p*	RD	*E* value	Standard care *n* = 3268	CBMCOC *n* = 830	RR 95% CI	*p*	RD	*E* value
Preterm birth (< 37 weeks)	672 (9.0)	42 (5.0)	057 (0.41, 0.77)	< 0.001	−4.0	2.90	268 (8.2)	42 (5.1)	0.60 (0.43, 0.82)	< 0.01	−3.1	2.72

*Note:* Data are *n* (%).

Abbreviations: CBMCOC, community‐based midwife continuity of care; CI, confident interval; RD, risk difference; RRs, risk ratio.

^a^ Adjusted for age, ethnicity, deprivation Index quintile, parity, previous preterm birth, social risk factors, Whooley positive, pre‐existing medical and mental conditions.

## 4 Discussion

### 4.1 Main Findings

This study found that women living in South London who were exposed to models of community‐based midwife continuity of care (CBMCOC), after adjustment were significantly less likely to have a preterm birth compared to women exposed to models of standard maternity care. They were also significantly more likely to have a spontaneous onset of labour, a spontaneous cephalic birth, 1st and 2nd degree tears, skin‐to‐skin contact established and home birth; and significantly less likely to miss appointments, use intrapartum analgesia/anaesthesia, or experience induction of labour, caesarean section, instrumental birth, episiotomy, blood loss of more than 500 mL, and postnatal admission longer than 7 days. It is probable that the reduction of blood loss over 500 mL reflects the lower caesarean birth rate in CBMCOC. There were no differences in stillbirth or neonatal death but infants of women in CBMCOC were significantly less likely to be low birthweight or small for gestational age. In terms of process outcomes, women in CBMCOC had significantly more referrals to mental health services, and fewer missed appointments compared to those in standard care. The likelihood of having a preterm birth was also significantly lower among Black, Asian and other ethnic minority groups, and women living in the most deprived areas exposed to CBMCOC, compared to similar groups of women exposed to standard care.

### 4.2 Interpretation

Many of our findings align with a previous metanalysis and individual studies. The recent 2024 update of a Cochrane review of randomised controlled trials (RCTs) of continuity of care models in participants from heterogeneous populations found women receiving these models were less likely to experience a caesarean birth, instrumental birth and episiotomy, and more likely to have a spontaneous vaginal birth and report a positive experience but no differences in preterm birth [15]. The review found no RCT of continuity of care targeting women from specific ethnic groups or disadvantaged backgrounds; however there is promising evidence from observational and synthesis data. Rayment‐Jones et al. [40], for example, found women with complex social factors who received community‐based caseload continuity of midwife care experienced more spontaneous births, water use for pain relief, skin‐to‐skin contact, early access to care and referral to support services. Follow‐up research highlighted the protective nature of community‐based midwife continuity of care in reducing preterm birth, low birthweight, and induction of labour, particularly for women with the highest level of social risk [22]. Various observational studies, including two smaller UK studies of caseload midwifery in inner‐city deprived and diverse communities have also shown decreases in preterm birth and caesarean birth [41, 42], and increases in spontaneous births, home births and Apgar scores of > 8 at 5 min [41]. Research with Australian Indigenous women showed that collaborative models of midwife continuity of care integrated with Indigenous governance, family services, and community‐based hubs improved antenatal attendance, reduced preterm birth and increased breastfeeding on discharge [21, 43]. An analysis of the Maternity Services Dataset covering 922 149 women in England found that midwife continuity of care increases the uptake of the first breast milk feed and may reduce stillbirth rates for Black women, providing key insights for future healthcare policy [44]. Finally, a recent systematic review of targeted health and social care interventions for women and infants impacted by health inequalities in high‐income countries found multi‐interventional approaches could enhance a targeted approach for at‐risk populations, in particular combining midwifery models of care with community‐centred approaches, to enhance accessibility, earlier engagement, increased attendance and improved outcomes [11]. Deprivation and ethnicity remain key drivers of inequalities in maternal health, and prevention strategies need to address social and structural determinants in areas of high deprivation and minority ethnicity groups [45].

Recent WHO, ICM and FIGO good practice recommendations highlighted that midwife continuity of care within existing, context‐appropriate care models, in primary as well as secondary care, is pivotal to delivering high‐quality care across the pregnancy continuum, prior to conception, through pregnancy and birth and beyond [46, 47]. Models or packages of care, such as CBMCOC, are complex healthcare interventions and understanding the mechanisms by which they influence outcomes, particularly among women from diverse and disadvantaged groups, is crucial. Synthesising findings from several realist reviews and studies of midwife continuity of care can offer a structured framework for this, and mechanisms can be grouped into three core themes: (1) the woman‐midwife partnership; (2) improved maternity pathways and processes and (3) enabling system resources. At the heart of the model is the woman‐midwife partnership, where relational continuity is key; this ongoing relationship engenders mutual trust and confidence between women and midwives. The response to this trust is that women feel safer, less anxious, more respected, and empowered, and this trusting environment facilitates the disclosure of sensitive social risk factors and eases women's perceptions of stigma or surveillance, particularly for those with social care involvement—this allows for the provision of practical and emotional support that enables women to become active participants in their care [48, 49, 50, 51]. Second, these models improve maternity pathways and processes. The known midwife acts as an effective care coordinator and advocate, helping women navigate what is often a fragmented and unfamiliar system, and this mechanism appears to be enhanced when care is based in the community. Midwives in community settings report better integration with local services, which in turn helps women build a wider support network [48, 50, 52]. Finally, the success of these models depends on enabling system resources. This includes not just organisational infrastructure and partnerships but also addressing the professional and systemic challenges that arise from changing models of care. Implementing continuity models disrupts established professional roles and power structures, which can create role ambiguity and conflict [49], thus overcoming these barriers requires strong leadership, a shared philosophy among providers, and clear policies to support midwives and ensure the model is sustainable [50, 51, 52].

Recent policy in England has focused on improving access to midwife continuity of care for women from ethnic minority groups and those living in deprived areas [16, 18]. Although current evidence shows that ethnicity is associated with socioeconomic deprivation, Black and minority ethnic women who are not socially deprived still experience worse outcomes than their white counterparts [53]. This could be due to area levels of deprivation being used rather than individual indicators, that overlook determinants of health such as wealth, social status, isolation and social capital [48]. Understanding the impact that these measures have on birth outcomes for ethnic minority groups will enable maternity providers to target women who are most at risk whilst avoiding stereotypical assumptions and racial profiling. It is important to recognise and build on the strengths of the most disadvantaged or marginalised in society. They are often the main target population for interventions, and find it hardest to access and engage with services [53], thus early involvement with representatives of ‘under‐served’ groups, intermediaries and advocates is crucial to ensure acceptability.

### 4.3 Strengths and Limitations

There is a paucity of research investigating the effect of community‐based midwife continuity of care models on preterm birth and other outcomes among women living in areas of social disadvantage and ethnic diversity in the UK. Our study used all eligible records from the eLIXIR maternity‐child data linkage and, uniquely, controlled for potential confounders through propensity score matching. This approach has more power than conventional regression modelling when, as in this study, the number of events is low and there are seven or fewer events per confounder, as this produces less biased and more precise estimates [54]. Using linked NHS records reduced selection bias, but data quality depended on clinicians' reporting, and limitations included potential misclassification of ethnicity, underreporting of sensitive issues (e.g., mental health), and missing contextual psychosocial information, which may have led to incomplete identification of risk factors [55]. All women in CBMCOC were successfully matched in a 1:4 ratio to women in standard care (much larger cohort). We grouped more than 20 ethnic groups into four main categories to make the analysis feasible and meaningful for our research purpose. While randomised trials can perfectly balance intervention and control groups on both measured and unmeasured unknown variables, propensity score matching can only account for the measured variables that were included in the analysis; this means there is a higher chance of unknown sources of bias remaining in the analysis. Following matching score adjustment, we found no baseline differences. Propensity score matching does have limitations given how it relies on the completeness and quality of the data available (e.g., accuracy), meaning unadjusted confounders may exist due to unmeasured factors influencing maternity model allocation [56]. *E*‐values for key associations exceeded the strength of known confounders in our models, which ranged in adjusted RRs from 0.00 to 2.72. For example, *E*‐values were 3.68 for planned caesarean birth, 2.90 for low birthweight and 4.31 for mental health referrals, suggesting that unmeasured confounding would need to be stronger than any measured covariate to nullify these findings [35]. Analysing women by their assigned care model enhances real‐world applicability and reduces bias, though it cannot account for variations in intervention fidelity; this is a recognised limitation when pragmatically evaluating complex healthcare interventions. We were unable to differentiate between spontaneous and medically indicated preterm births; and this is an important distinction, as the underlying causal pathways and the potential impact of the care model may differ for each; future research should aim to capture this level of detail to better elucidate the potential mechanisms of action.

## 5 Conclusion

Our findings support the current policy drive to increase continuity of midwife care, and that adding community‐based care may further improve outcomes for women at increased risk of health inequalities. However, future trials should evaluate the effectiveness, implementation, scale‐up, and cost of these models to understand their real‐world impact, and how they may benefit ‘at risk’ women and babies throughout their life course by improving short‐and long‐term health outcomes and social determinants, and contributing to mitigate inequalities in maternal and newborn health in the UK.

## Author Contributions

C.F.T. conceptualization, data curation, investigation, methodology, project administration, visualisation, writing (original draft), writing (review and editing). S.B. conceptualization, data curation, formal analysis, methodology, software, visualisation, writing (original draft), writing (review and editing). Z.K. conceptualization, methodology, writing (review and editing). Z.V. conceptualization, methodology, writing (review and editing). M.N. conceptualization, writing (review and editing). P.T.S. conceptualization, methodology, supervision, writing (review and editing). H.R.‐J. conceptualization, methodology, visualisation, writing (review and editing). J.S. funding acquisition, conceptualization, methodology, supervision, validation, visualisation, writing (review and editing). A.E. funding acquisition, conceptualization, methodology, supervision, visualisation, validation, writing (review and editing).

## Funding

J.S., C.F.T., A.E., S.B., Z.V., Z.K. and this research was funded by the National Institute for Health Research (NIHR) Applied Research Collaboration South London (NIHR ARC South London) at King's College Hospital NHS Foundation Trust. The views expressed are those of the author[s] and not necessarily those of the NIHR or the Department of Health and Social Care. The work was supported by the Early Life Cross Linkage in Research, Born in South London (eLIXIR‐BiSL) Partnership developed by an MRC Partnership Grant (MR/P003060/1) and the MRC Longitudinal Population Study Grant (MR/X009742/1). The eLIXIR platform is also part‐supported by the National Institute for Health Research (NIHR) Biomedical Research Centre at the South London and Maudsley NHS Foundation Trust and King's College London. HRJ is funded by a NIHR Advanced Fellowship (NIHR 303183).

## Ethics Statement

Ethical approval eLIXIR is granted by the Oxford Central Research Ethics Committee (23/SC/0116). The Health Research Authority Confidentiality Advisory Group (HRA CAG Ref: 18/CAG/0040) provided approval under Section 251 (s251) of the NHS Act (2006). This study was also approved by the eLIXIR Oversight Committee (RAF: DL021R).

## Consent

The authors have nothing to report.

## Conflicts of Interest

The authors declare no conflicts of interest.

## Supporting information


**Table S1:** bjo70101‐sup‐0001‐TableS1.docx.

## Data Availability

The data accessed by eLIXIR remains within an NHS firewall and governance is provided by the eLIXIR Oversight Committee reporting to relevant information governance clinical leads. Subject to these conditions, data access is encouraged, and those interested should contact the eLIXIR Chief Investigator (Professor Lucilla Poston; lucilla.poston@kcl.ac.uk).
