# Supplementary material for: The Impact of Community‐Based Midwife Continuity of Care Models for Women Living in Areas of Social Disadvantage and Ethnic Diversity in the United Kingdom: A Prospective Cohort Study
Source: BJOG. 2026 Jan 20;133(5):958–73. doi: 10.1111/1471-0528.70101 (PMC12972849; doi:10.1111/1471-0528.70101)
Supplement: Supplementary file 1 — Table S1: bjo70101‐sup‐0001‐TableS1.docx. [file BJO-133-958-s001.docx]

| **Table S1: Exposure and outcome measures and corresponding database variables** | | |
| --- | --- | --- |
| **Exposure** | **Dataset** | **How identified in database?** |
| Name of team | DS1 Booking dataset | Teamname  Exposure: LEAP, ACORN, WILLOW, OLIVE, GROVE, ELECTRIC, JUNIPER, BIRCH |
| **Outcome** | **Data set** | **How identified in database?** |
| Pre-term birth | DS2 Intrapartum and postnatal care | GestationAtDeliveryWeeks <37 |
| Onset of labour | DS2 Intrapartum and postnatal care | OnsetOfLabour |
| Analgesia | DS2 Intrapartum and postnatal care | AnalgesiaAndAnaesthesiaSummary, free text search for “epidural” and “spinal” |
| Type of birth | DS2 Intrapartum and postnatal care | TypeOfDelivery |
| Perineal status | DS2 Intrapartum and postnatal care | PerinealTrauma |
| Obstetric haemorrhage | DS2 Intrapartum and postnatal care | TotalBloodLoss >499ml |
| Place of birth | DS2 Intrapartum and postnatal care | ActualPlaceofBirth not including “not recorded” |
| Skin to skin established | DS2 Intrapartum and postnatal care | SkinToSkinEstablished |
| Prolonged length of postnatal stay | DS2 Intrapartum and Postnatal Information | Definition: Indicates whether the postpartum hospital stay exceeded expected duration based on delivery mode. Criteria: Vaginal delivery: Length of stay > 48 hours ; Caesarean delivery: Length of stay > 4 days |
| Birth outcome | DS2 Intrapartum and postnatal care | FinalBirthOutcome |
| Five minute Apgar score | DS2 Intrapartum and postnatal care | APGARscore5Minutes |
| First feed method | DS2 Intrapartum and postnatal care | FirstFeedMethod |
| Admission to neonatal unit | DS2 Intrapartum and postnatal care | AdmittedToNeonatalUnit |
| Late Booking | DS1 Booking dataset | LateBookingReason not including “Transfer of care (clinical reason)”, “Transfer of care (non-clinical reason)”, “Transfer from private care” |
| Missed appointment | DS4 Antenatal appointment | Attended |
| Antenatal admission | DS4 Antenatal appointment | Attended, AppointmentReason |
| Smoking referral | DS1 Booking dataset | SmokingReferral |
| Mental health care referral | CRIS – Maternal Mental health | Pregnancy ePJS =1 (indicative of a referral to secondary mental health services) |
| Child protection referral | DS8 Other social issues | UnbornChildProtection |
